# Environmental Shaping Suitable Habitats and Quality of *Lonicera macranthoides* Hand.−Mazz.: Insights from MaxEnt, HPLC, Chemometrics, and Gene Expression Analysis

**DOI:** 10.3390/plants15101425

**Published:** 2026-05-07

**Authors:** Nan Xu, Canfeng Long, Meixin Zhou, Weijia Wang, Qiang Zeng, Yingying Shen, Pan Wu, Liqun Rao, Guoping Peng, Qiming Wang

**Affiliations:** 1 College of Bioscience and Biotechnology, Hunan Agricultural University, Changsha 410128, China; x449734271@163.com (N.X.); 18373217234@163.com (C.L.); xmz040524@163.com (M.Z.); godfengl@gmail.com (W.W.); 15616705520@163.com (Q.Z.); syy19918318242@163.com (Y.S.); w011227@stu.hunau.edu.cn (P.W.); raoliqun@163.com (L.R.); pgphh@163.com (G.P.); 2 Yuelushan Laboratory, Changsha 410128, China; 3 Hunan Engineering Laboratory for Good Agricultural Practice and Comprehensive Utilization of Famous−Region Medicinal Plants, Hunan Agricultural University, Changsha 410128, China

**Keywords:** MaxEnt, climate change, combined stress, suitable habitat, chemometrics, HPLC

## Abstract

*Lonicera macranthoides* Hand.−Mazz. is a valuable medicinal plant in China and is used worldwide. This study aimed to predict its suitable habitats in China using the MaxEnt model, and to assess the effects of environmental variables on indicator ingredients (chlorogenic acid, macranthoidin B, and dipsacoside B) via HPLC and chemometrics. Furthermore, to explore the molecular mechanisms underlying environment−quality relationships, preliminary indoor versus outdoor stress experiments were conducted, analyzing the expression of chlorogenic acid biosynthetic genes using qRT−PCR. The results showed that precipitation of the driest month was the most influential variable affecting distribution. Currently, suitable areas are mainly located between 21° N and 33° N. During the Last Glacial Maximum (LGM), habitats were more expansive, whereas they contracted during the Mid−Holocene (MH). Future projections indicated habitat loss under the SSP585 scenario, which was partially mitigated under the SSP126 scenario by 2090 S. Higher contents of chlorogenic acid and saponins were found in suitable habitats and were associated with soil, altitude, and precipitation. Notably, outdoor combined stress (low temperature and low sunshine) significantly regulated the expression of *LmPAL*, *LmCHS*, *LmCHI*, *LmC4H*, *LmCCoAOMT*, and *LmANS*. This study serves as a scientific basis for the conservation, sustainable cultivation, and stress−oriented breeding of *L. macranthoides* in China.

## 1. Introduction

*Lonicera macranthoides* Hand.−Mazz. is a popular medicinal plant in southern China due to the economic value of its dried flowers [1,2]. Modern pharmacological studies have demonstrated that *L. macranthoides* has various pharmacological effects, such as immunomodulatory activity, anti−inflammatory and antibacterial properties, and hypoglycemic and lipid−lowering effects [3,4,5]. Currently, *L. macranthoides* wild resources are relatively abundant but exhibit uneven quality; consequently, the source of medicinal materials is derived mainly from cultivated sources [6,7]. The main cultivated areas of *L. macranthoides* are in Hunan, Guizhou, Chongqing, Sichuan, Yunnan, and other places. However, different geographical units can impact the chemical components of medicinal plants, thereby affecting their quality [8,9,10]. In the Pharmacopoeia of the People’s Republic of China (PPRC), chlorogenic acid, macranthoidin B, and dipsacoside B serve as indicator ingredients for assessing the quality of *L. macranthoides* [6]. Regarding medicinal material quality, based on differences in the content of nine bioactive compounds, Chongqing, Hunan, and Guizhou were identified as the most suitable provinces for *L. macranthoides*, while Sichuan exhibited relatively lower quality [7].

Climate change is a critical driver of variations in medicinal plant ingredients and impacts on their habitats and sustainable development [11,12,13,14]. Species distribution models (SDMs) are tools that combine species distribution information with environmental factor data to predict species’ geographical distribution and can be employed to address climate change [15,16,17]. Currently, the MaxEnt model can assess species’ potential suitable habitat distribution and analyze the correlation between the potential distribution and environmental variables [18,19,20,21]. Compared with models such as Bioclim, DOMAIN, GLM, GAM, and MaxLike, MaxEnt has unique advantages in predicting the distribution for small sample sizes and little−known species because it utilizes presence−only data and can capture complex environmental responses [22,23,24,25]. When medicinal plant distribution data are limited, the MaxEnt model has higher prediction accuracy and a more robust capture of nonlinear relationships [15,26]. Studies have shown that specific environmental variables can significantly enhance metabolite accumulation in medicinal plants [11,27,28,29]. Unfortunately, the mechanism by which climate change affects secondary metabolites remains unclear. In recent years, research on *L. macranthoides* has focused on chemical composition analysis [30], pharmacology [31,32], or omics approaches to explore key genes and metabolites [33,34,35,36], with limited research on species distribution [7]. Furthermore, plants in their natural habitats are often subjected to combined stress, such as low temperature, drought, or fluctuating light, which trigger complex molecular and metabolic responses [37,38]. Understanding how *L. macranthoides* perceives and integrates these environmental signals at the molecular level is crucial for linking habitat suitability to final product quality. Fortunately, MaxEnt, HPLC, chemometric approaches, and qRT−PCR can be effectively employed to evaluate the impact of environmental variables on secondary metabolites in medicinal plants, and this integrated approach has been successfully applied to numerous medicinal plant species [15,26,39]. To our knowledge, no studies have comprehensively used the MaxEnt model to assess the habitat distribution and quality of *L. macranthoides* in China, nor have explored the underlying molecular mechanisms by which combined stress influences its quality.

The primary purposes of this study are as follows: (1) to construct a MaxEnt model to predict the distribution of *L. macranthoides*, delineate suitable habitats, and identify the key environmental variables influencing its distribution; (2) to evaluate the effects of environmental on the quality of *L. macranthoides* using HPLC and chemometrics; (3) to preliminarily determine the transcriptional changes in *L. macranthoides* grown in indoor and outdoor environments using qRT−PCR. This study will fill the research gap regarding the distribution of *L. macranthoides* in China and how suitable habitats affect its compounds; thereby establishing a foundational theoretical framework for the conservation, sustainable utilization, transplantation, and cultivation of *L. macranthoides*.

## 2. Results

### 2.1. Evaluation of Model Accuracy

**Figure 1:**
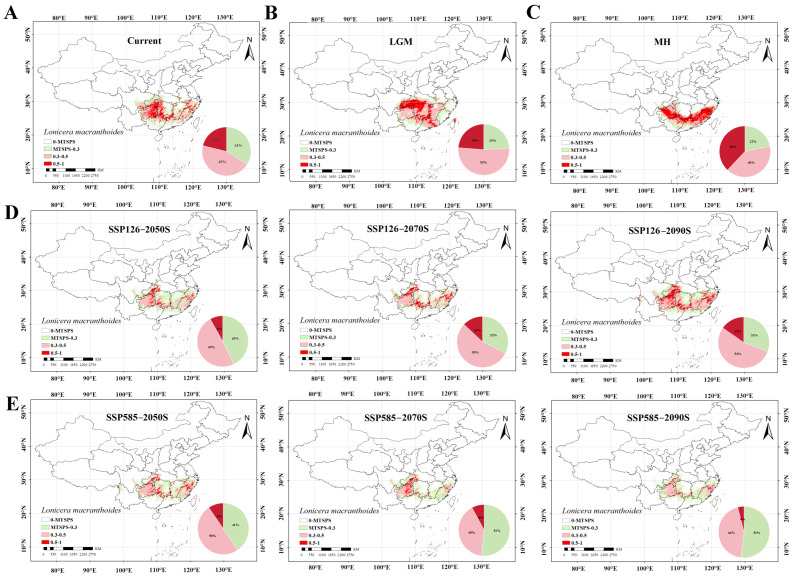
The range of suitable habitats for *L. macranthoides* under different climate scenarios. (**A**) Current; (**B**) Last glacial maximum (LGM); (**C**) Middle Holocene (MH); (**D**) SSP126—2050 S, 2070S, 2090S; (**E**) SSP585—2050 S, 2070S, 2090S.

Our study collected 34 samples from China’s representative producing areas (Table S1) and 329 occurrence records from a public database (Figure S1). The MaxEnt model was constructed using the occurrence records. We utilized AUC and TSS values to measure the accuracy of the MaxEnt model. AUC > 0.9 and TSS > 0.8 were considered highly accurate predictions. We selected 21 environmental variables, which effectively mitigated overfitting and improved the model’s prediction accuracy. We found that each mean AUC value was >0.95, and each TSS value was between 0.842 and 0.856 (Figure 1, Table S2). Thus, the MaxEnt model is highly accurate in predicting the suitable habitat distribution of *L. macranthoides*.

### 2.2. Analysis of Environmental Variables

In this study, *L. macranthoides* distribution was influenced by eight key variables, including Bio_14 (precipitation of driest month), Ai _v3_yr (the global aridity index), Elev (elevation), Bio_4 (temperature seasonality), T_caco3 (topsoil carbonate or lime content), Slope (slope), UVB4 (mean UV−B of lowest month), and Gm_lc_v3 (land cover) (Table S3). The combined percent contribution and permutation importance of the above eight variables reached 93.3% and 57.9%, respectively. Bio_14 exhibited the highest individual contribution. Furthermore, Ai _v3_yr, Bio_14, and Bio_6 demonstrated higher weight shares (Figure S3). Bio_14 had the highest weight share, which was significant for *L. macranthoides*.

The MaxEnt model also generated response curves of *L. macranthoides* in relation to environmental factors. Except for T_caco3 and Gm_lc_v3, key environmental variables exhibited a single−peaked normal distribution in their response curves (Figure S4). These curves determined the ranges of suitable environmental variables for the potential distribution of *L. macranthoides*. The ranges and optimum values of suitable environmental variables for the distribution were as follows (Table 1): The precipitation of the driest month ranged from 0 to 190 mm, with an optimal precipitation of 50.31 mm. The global aridity index was 54–54,506, with an optimal index of 17,740.01. The elevation ranged from −79 to 6520 m, with an optimal elevation of 512.27 m. The temperature seasonality ranged from 307.041 to 1735.564, with an optimum of 740.17. The topsoil carbonate or lime content ranged from 0 to 15%, with an optimal content of 7.18%. The slope ranged from 0 to 11.853°, with an optimal slope of 2.17°. The mean UV−B of the lowest month ranged from 129.596 to 3000.134, with an optimal of 1337.52 J·m^2^·day^−1^. The land cover ranged from 1 to 20, with an optimum of 12.51.

### 2.3. Analysis of Distribution Under Current Climate Scenarios

Under current climatic scenarios, *L. macranthoides* was primarily distributed in southern China, ranging from 21° N to 33° N, particularly between 23° N and 31° N. The suitable habitat area (defined as the total area of high– and medium–suitable habitat) of *L. macranthoides* was 659.307 × 10^3^ km^2^. The area of high–suitable habitats was 207.78 × 10^3^ km^2^. These were primarily distributed in eastern Guizhou, southwestern Hunan, southeastern Chongqing, southwestern Hubei, northern Guangxi, northern Guangdong, northeastern Fujian, southern Zhejiang, the eastern Sichuan Basin, and other regions. The medium–suitable habitat area was 451.52 × 10^3^ km^2^. These were mainly distributed in western Guizhou, western Hunan, northern Guangxi, northern Guangdong, Chongqing, Fujian, southern Jiangxi, western Zhejiang, southern Anhui, and the eastern Sichuan Basin, as well as scattered areas in Shaanxi, Taiwan, and Yunnan. Furthermore, the area designated as low–suitable habitat was 329.44 × 10^3^ km^2^. Compared with high−suitable habitats, medium– and low–suitable habitats were more diffusely distributed, mainly extending from the area of high–suitable habitats to surrounding suitable habitats (Figure 1A, Table S4).

### 2.4. Analysis of Distribution Under Past Climate Scenarios

Under past climate scenarios, the suitable habitat of *L. macranthoides* during the LGM period was primarily distributed in Hunan, Guizhou, northern Guangxi, Chongqing, Guangdong, Jiangxi, southwestern Hubei, eastern Sichuan, southern Henan, and northeastern Taiwan, as well as scattered areas in Tibet, Zhejiang, Fujian, and Yunnan. Compared with the current period, the suitable habitat area was larger in the LGM period, covering 849.23 × 10^3^ km^2^, representing an increase of 189.93 × 10^3^ km^2^ (Figure 1B and Figure S5, Table S4).

The suitable habitat of *L. macranthoides* during the MH period mainly covered Guizhou, southeastern Chongqing, western and southern Hunan, the southeastern Sichuan Basin, southwestern Hubei, northern Guangxi, northern Guangdong, Fujian, southern Jiangxi, southwestern Zhejiang, and some areas in Yunnan and Taiwan. Compared with the LGM period, the habitat in the MH period expanded significantly southward, eastward, and westward, showing a zonal distribution. However, the area of suitable habitats was 613.28 × 10^3^ km^2^, a decrease of 235.42 × 10^3^ km^2^. This suggested that the suitable habitat of *L. macranthoides* was contracted in the MH period (Figure 1C and Figure S5, Table S4).

### 2.5. Analysis of Distribution Under Future Climate Scenarios

Under the SSP126 climate scenario, the suitable habitat area of *L. macranthoides* in the 2041–2060 (2050 S), 2061–2080 (2070 S), and 2081–2100 (2090 S) periods showed a trend of initially decreasing and then increasing, compared with the current period. During the SSP126−2050 S period, the suitable habitat area was 503.10 × 10^3^ km^2^, with high– and medium–suitable habitat areas of 71.52 × 10^3^ km^2^ and 431.58 × 10^3^ km^2^, respectively. The area of high–suitable habitat decreased by 136.16 × 10^3^ km^2^ relative to the current period, mainly in Guizhou, Hunan, Guangxi, Chongqing, and Zhejiang. During the SSP126−2070 S period, the high–, medium–, and low–suitable habitat areas were 71.51 × 10^3^ km^2^, 418.36 × 10^3^ km^2^, and 245.75 × 10^3^ km^2^, respectively. The suitable habitat area was equivalent to that of the SSP126−2050 S period. During the SSP126−2090 S period, the suitable habitat area was 766.14 × 10^3^ km^2^, with high– and medium–suitable habitat areas of 165.77 × 10^3^ km^2^ and 600.37 × 10^3^ km^2^, respectively. Interestingly, during the SSP126−2090 S period, the suitable habitat area largely returned to the level observed in the LGM period and was higher than that observed in the current period (Figure 1D and Figure S5, Table S4).

Under the SSP585 climate scenario, compared with the current period, the suitable habitat of *L. macranthoides* exhibited a continuous decreasing trend in the future. During the SSP585−2050 S period, the suitable habitat area was 503.59 × 10^3^ km^2^, and the high– and medium–suitable habitat areas were 81.28 × 10^3^ km^2^ and 422.31 × 10^3^ km^2^, respectively. The suitable habitat area decreased by 155.71 × 10^3^ km^2^ compared with the current period, with the main decrease occurring in Hunan. During the SSP585−2070S period, the suitable habitat area was 346.55 × 10^3^ km^2^, and the areas of high and medium–suitable habitats were 56.76 × 10^3^ km^2^ and 289.79 × 10^3^ km^2^, respectively. During the SSP585−2090S period, the suitable habitat area was 351.06 × 10^3^ km^2^, and the high– and medium–suitable habitat areas were 29.14 × 10^3^ km^2^ and 321.92 × 10^3^ km^2^, respectively. Unfortunately, the reduction in suitable habitat area from the SSP585—2050 S period was not alleviated (Figure 1E and Figure S5, Table S4).

### 2.6. Pattern Changes in Distribution Areas Across Different Periods

**Figure 2:**
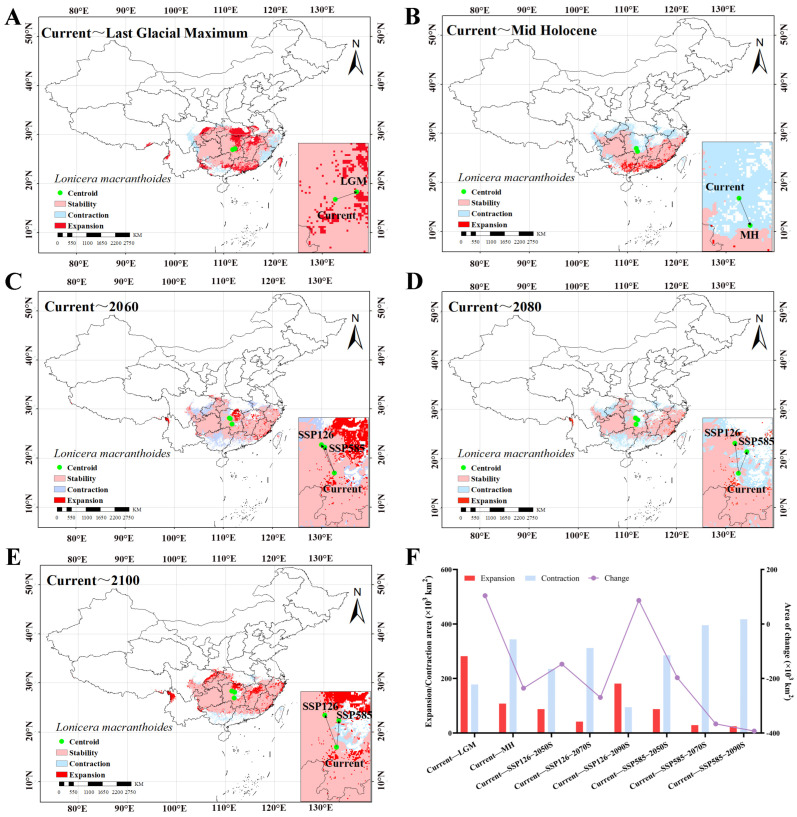
Dynamic changes in the distribution area for *L. macranthoides* and the transfer path of the centroids under different climate scenarios (compared with the current climate). (**A**) LGM; (**B**) MH; (**C**) 2050 S, current~2060; (**D**) 2070 S, current~2080; (**E**) 2090 S, current~2100; (**F**) Dynamic changes in the distribution area.

The geometric centroid of the current distribution area was located in Qidong County, Hunan Province (111.84° E, 26.89° N). Compared with the current period, the distribution area during the LGM expanded northward and southward, while contracting in the west and east. The total distribution area increased by 103.85 × 10^3^ km^2^. The centroid during this period was situated northeast of the current centroid, at a distance of 50.25 km (Figure 2A,F, Table S5). However, the distribution area during the MH period shifted southward, with a total decrease of 236.06 × 10^3^ km^2^. The centroid moved 70.84 km toward the southeast (Figure 2B,F, Table S5). Collectively, from past periods to the current period, the distribution centroid exhibited a pattern of southward movement followed by northward movement in response to temperature variations (Figure S6).

By 2050 S, the average expanded area under both scenarios was 87.33 × 10^3^ km^2^, while the average contracted area was 259.81 × 10^3^ km^2^, resulting in a net reduction of 172.48 × 10^3^ km^2^. During this period, the centroids under both scenarios were located northwest of the current centroid, at distances of 146.96 km (SSP126) and 131.22 km (SSP585), respectively (Figure 2C,F, Table S5). By 2070 S, the average area under both scenarios continued to decrease, with a net reduction of 318.39 × 10^3^ km^2^. The centroids of both scenarios were located north of the current centroid, at distances of 110.08 km (SSP126) and 145.49 km (SSP585), respectively (Figure 2D,F, Table S5). Interestingly, by the 2090S, the two scenarios presented contrasting extremes: SSP126 demonstrated a more positive trend with a net area increase of 86.61 × 10^3^ km^2^, while SSP585 continued to decline with a net reduction of 392.66 × 10^3^ km^2^. Both centroids shifted northward and were located 161.46 km (SSP126) and 127.07 km (SSP585) from the current centroid, respectively (Figure 2E,F, Table S4). Overall, under both future scenarios, the distribution centroid of *L. macranthoides* is projected to migrate toward colder northern regions; with increasing CO_2_ emissions, the centroid is expected to shift further northward (Figure S6).

### 2.7. Analysis of the Content of Indicator Ingredients

**Figure 3:**
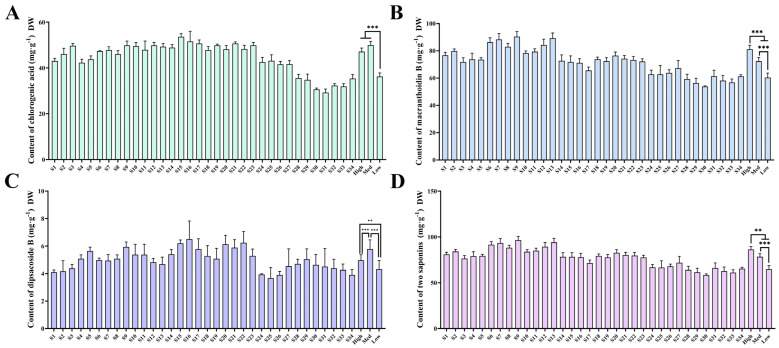
Content of chlorogenic acid (**A**), macranthoidin B (**B**), dipsacoside B (**C**), and two saponins (**D**) in *L. macranthoides* from different suitable habitats. Hig (high–suitable habitats) was the average content of S1–S13. Med (medium–suitable habitats) was the average content of S14–S23. Low (low–suitable habitats) was the average content of S24–S34. The statistical analysis was defined as ** *p* < 0.01, and *** *p* < 0.001.

This study investigated the differences in the contents of indicator ingredients of *L. macranthoides* from different suitable habitats. The chlorogenic acid content in all samples reached 2% of the quality evaluation standard for herbs specified by the PPRC. In addition, the sum of macranthoidin B and dipsacoside B in all samples met the PPRC requirement of 5%. The average chlorogenic acid and macranthoidin B contents in low–suitable habitats were significantly lower than those in suitable habitats. Meanwhile, the content of dipsacoside B was significantly lower in low–suitable habitats than in medium–suitable habitats. Among these samples, the highest chlorogenic acid and dipsacoside B contents were found in Zunyi, at 53.61 mg·g^−1^ and 6.50 mg·g^−1^, respectively, while the highest macranthoidin B content was observed in Huaihua, at 90.43 mg·g^−1^. The lowest chlorogenic acid and macranthoidin B contents were observed in Changsha, at 29.23 mg·g^−1^ and 53.77 mg·g^−1^, respectively, and the lowest dipsacoside B level was found in Zhaotong, which was 3.67 mg·g^−1^. The sum of the two saponins increased in the suitable habitats. The chlorogenic acid and saponin contents of *L. macranthoides* in high– and medium–suitable habitats were higher than those in low–suitable habitats, indicating that herbal quality in suitable habitats was superior to that in low–suitable habitats (Figure 3).

### 2.8. HCA, PCA, and Correlation Analysis

**Figure 4:**
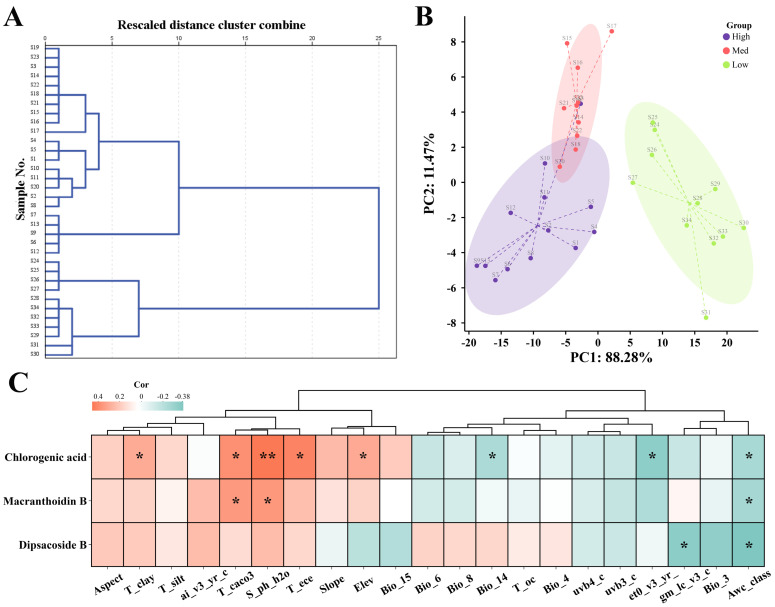
A multivariate data analysis of 34 *L. macranthoides* samples in different suitable habitats. (**A**) HCA analysis. (**B**) PCA score plot analysis. (**C**) Heatmap of Pearson’s correlation between *L. macranthoides* indicator ingredients content and environmental factors. The statistical analysis was defined as * *p* < 0.05 and ** *p* < 0.01.

To explore the effect of habitat suitability on *L. macranthoides*, HCA and PCA were employed to analyze the HPLC data of 34 samples from three suitability categories (high–, medium–, and low–suitable habitats). The HCA results showed that the 34 samples were clearly divided into two categories: those from low–suitable habitats were grouped in the first category. Samples from suitable habitats were classified into the second category. This result was closely related to significant environmental differences (Figure 4A). The PCA results showed that the first two principal components explained 88.28% and 11.47% of the total variance, respectively. All samples were clearly divided into three categories, especially the samples from low–suitable habitat areas showed significant differences (Figure 4B).

To investigate the factors leading to changes in indicator ingredients in *L. macranthoides*, correlation analysis between indicator ingredients and environmental variables was performed. Correlation analysis showed that environmental factors had a significant impact on the accumulation of chlorogenic acid, macranthoidin B, and dipsacoside B. Specifically, the level of chlorogenic acid was positively correlated with T_clay (topsoil clay content), T_caco3, S_ph_h2o (substratesoil pH), T_ece (topsoil electroconductibility), Elev, and negatively correlated with Bio_14, Et0_v3_vr (standard deviation of annual potential Evapo−transpiration), Awc_class (soil available water content). The accumulation of macranthoidin B and dipsacoside B was mainly related to T_caco3, S_ph_h2o, Gm_lc_v3 (land cover), and Awc_class. These results indicated that the accumulation of chlorogenic acid was mainly affected by soil, altitude, and precipitation, and its saponin content was mainly related to soil factors (Figure 4C).

### 2.9. Gene Expression Patterns Under Indoor and Outdoor Environments

**Figure 5:**
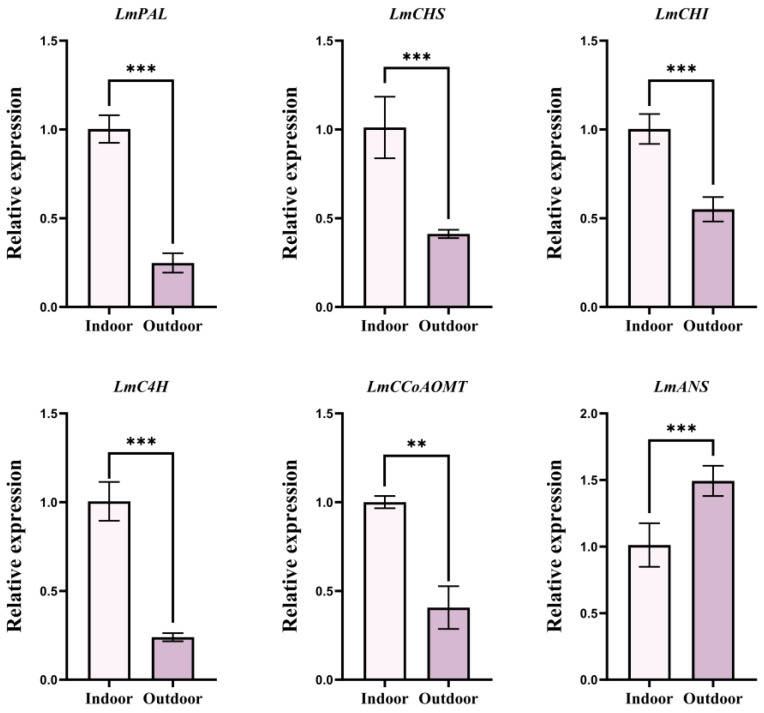
Effects of indoor and outdoor environments on the relative expression levels of chlorogenic acid–related genes in *L. macranthoides*. The statistical analysis was defined as ** *p* < 0.01 and *** *p* < 0.001.

The distribution of *L. macranthoides* was closely related to environmental factors such as temperature seasonality and mean UV−B of the lowest month. To further explore the relationship between chlorogenic acid accumulation in *L. macranthoides* and temperature and light, the expression of chlorogenic acid–related genes under indoor and outdoor conditions was assessed. Compared with the indoor environment (25 °C, 20,000 lux), the expression of *LmPAL*, *LmCHS*, *LmCHI*, *LmC4H*, and *LmCCoAOMT* in the outdoor environment (8 °C, low sunshine) of *L. macranthoides* was significantly decreased, and the expression of *LmANS* was significantly increased (Figure 5. These results indicated that the combined stress of low temperature and low sunshine significantly affected the transcriptional levels of phenylpropanoid pathway–related genes in *L. macranthoides*, suggesting that environmental stress plays a key role in regulating secondary metabolism at the molecular level.

## 3. Discussion

### 3.1. Environmental Impact on the Distribution and Quality of L. macranthoides

Few studies have investigated the distribution of *L. macranthoides*, and none have identified the environmental variables causing differences in its distribution [7]. It is a perennial medicinal liana plant that prefers warm, humid, and sunny environments [6]. However, due to its well–developed root system, it is also highly adaptable and exhibits drought and salt tolerance [3,40]. This finding indicated that precipitation of the driest month, the global aridity index, elevation, temperature seasonality, topsoil carbonate or lime content, slope, mean UV−B of the lowest month, and land cover were the primary factors affecting the distribution of *L. macranthoides* in China, with precipitation playing a prominent role. Climatic variables such as precipitation appear more critical for *L. macranthoides* than soil and topographic variables. This is consistent with previous studies on *Dendrobium* [41], *Litsea cubeba* [17], *Hylomecon japonica* [42], and *Pseudostellaria heterophylla* [43]. This may be attributed to the fact that precipitation is directly linked to plant water requirements [44,45]. Therefore, precipitation is a critical limiting factor for the distribution of *L. macranthoides*.

Furthermore, the contribution rate of precipitation of the driest month reached 64.2%, indicating that *L. macranthoides* can tolerate drought stress in adapted environments. The higher contribution of the global aridity index (reaching 13.6%) further confirms this observation. However, it is still necessary to ensure an adequate water supply. Some studies have shown that plant distribution is related to drought stress adaptation [46,47]. Drought stress can pose a significant threat to plants, disrupting their osmotic balance and cell structure, thereby impairing physiological functions [48]. In addition, our results have shown that the topographic variables (slope and elevation) are key factors affecting the distribution of *L. macranthoides*. This is because slope and elevation are closely related to precipitation and temperature [49]. Studies have shown that slope influences water redistribution [50,51], light resource allocation [52,53], and soil structure [54], thereby impacting plant growth. In contrast, elevation mainly affects plant light availability and temperature adaptability [52,55]. It is noteworthy that UVB4 also demonstrated a considerable contribution and importance in our study, indicating that *L. macranthoides* requires a certain intensity of ultraviolet radiation, which aligns with its heliophilic nature.

The type and quantity of chemical ingredients primarily influence the quality of medicinal plants [56,57]. The results showed differences in the content of indicator ingredients across different suitable areas of *L. macranthoides*. All indicator ingredients exhibited higher contents in high– and medium–suitable habitats and lower contents in low–suitable habitats. This result is consistent with previous studies, demonstrating that higher indicator ingredient contents in suitable habitats contribute to superior quality [7]. The differences in content in different suitable areas were verified by HCA and PCA analysis. Thus, a suitable environment is paramount for active ingredient accumulation [58,59]. The contents of indicator ingredients were lowest in low–suitable habitats, confirming the pivotal role of suitable habitats in active ingredient accumulation. This also indicated that *L. macranthoides* grown in Guizhou, Hunan, Chongqing, and other regions tends to accumulate active ingredients more readily, which is consistent with previous studies [7]. In addition, chlorogenic acid and dipsacoside B contents were significantly enriched in medium–suitable habitats, indicating that appropriate environmental stress may promote indicator ingredient enrichment in medicinal plants [60]. This phenomenon has also been observed in other medicinal plants. For example, short–term cold stress increased the concentration of secondary metabolites of *Panax ginseng* [61]. The combination of shading and potassium application regulated the accumulation of medicinal substances in *Fritillaria thunbergii* miq [62]. Moderate phosphorus deficiency promoted the accumulation of alkaloids and polysaccharides in *Dendrobium officinale* [63]. We further employed correlation analysis to evaluate the influence of 21 environmental variables on the quality of *L. macranthoides*. The contents of chlorogenic acid and saponins are closely related to strong compressive resistance [1,6]. Our results showed that the environment had a significant effect on the accumulation of chlorogenic acid and saponins, with key factors including soil, altitude, precipitation, and land cover. Studies have shown that precipitation, altitude, and temperature are also key environmental factors affecting the potential distribution and quality of *Arismatis rhizome* [64]. This result coincides with the actual cultivation process, especially regarding soil clay content. This study provides accurate environmental data to support site selection, cultivation, and yield improvement of *L. macranthoides*.

Although the above results provided valuable insights, the underlying molecular mechanisms by which combined stress regulates the accumulation of indicator ingredients remain largely unexplored. Therefore, we conducted a preliminary experiment comparing *L. macranthoides* plants grown indoors (25 °C, 20,000 lux) and outdoors (low– temperature and low sunshine) in December. qRT−PCR analysis showed that the transcript levels of key biosynthetic genes involved in the phenylpropanoid pathway, including *LmPAL*, *LmCHS*, *LmCHI*, *LmC4H*, and *LmCCoAOMT*, were substantially downregulated in outdoor samples, except for *LmANS*. The upregulated expression of *LmANS* was mainly due to the possibility that combined stress may inhibit transcription factors that initiate the upstream phenylpropanoid pathway and may activate the specific regulatory network driving the anthocyanin branch, especially the MYB−bHLH−WD40 complex [65,66,67,68]. These genes are known to play critical roles in chlorogenic acid biosynthesis in *L. macranthoides* [1,36,69]. The changes in gene expression indicated that the combined stress of outdoor low temperature and low sunshine likely impacted the transcriptional regulation of the phenylpropanoid pathway. Therefore, the synthesis of secondary metabolites such as chlorogenic acid also requires a suitable environment.

### 3.2. The Suitable Distribution and Spatial Variation of L. macranthoides

We established past, current, and future suitable distribution models for *L. macranthoides* in China based on the MaxEnt model. The simulation results are consistent with the documented distribution area under the current climate scenario [1,6]. We projected the LGM and MH periods to understand the spatial distribution changes of *L. macranthoides* in the past periods. This study found that the distribution area of *L. macranthoides* during the LGM period increased by 103.85 × 10^3^ km^2^. This was attributed to colder temperatures during the LGM period, with the global average temperature being approximately 6.1 °C lower than the 20th–century average. High–latitude regions exhibited heightened sensitivity to climate change, with lower temperatures than low–latitude regions [70,71]. However, the distribution area of *L. macranthoides* decreased by 236.06 × 10^3^ km^2^ during the MH period. This may be attributed to the fact that *L. macranthoides* requires suitable temperature conditions, whereas the excessively high temperatures during the MH period adversely affected its distribution area [72]. Meanwhile, this also accounts for the southward shift in the distribution centroid, followed by northward movement. Therefore, temperature is a pivotal factor influencing the distribution of *L. macranthoides*.

Under the two future climate scenarios, SSP126 and SSP585, the suitable distribution of *L. macranthoides* is projected to change differently. The suitable habitats were projected to decrease initially and then exceed the current level, from the SSP126−2050 S to the SSP126−2090 S. However, from the SSP585−2050 S to the SSP585−2090 S, the suitable habitat area was projected to continue decreasing without recovery. This indicated that environmental changes, especially precipitation and temperature, will impact the area of high–suitable habitats for *L. macranthoides* over the next 80 years. Fortunately, under the SSP126 future climate scenarios, suitable habitats in the 2090 S were projected to be higher than those under current scenarios, suggesting environmental improvement by the 2090 S. Interestingly, in the most pessimistic scenario, suitable habitats in 2090 S were significantly lower than in the most optimistic scenario. This may be related to the preference of *L. macranthoides* for moderate temperatures, as excessively high temperatures are detrimental. Similar results were observed in *Lysimachia christinae* [73], *Alpinia officinarum* [74], and *Eucommia ulmoides* [75], suggesting that increased climate variability, rising temperature, and unstable precipitation patterns under the SSP585 climate scenario will lead to large–scale habitat contraction for these habitats. Studies have shown that environmental changes can influence the distribution of many medicinal plants in relation to temperature and plant tolerance [11,59,76]. In addition, against the background of global warming, the general climate change trend in China involves warming in the north and cooling in the south, and most species are exhibiting northward migrating [77]. Meanwhile, the suitable habitat of *L. macranthoides* is projected to migrate northward. We speculate that *L. macranthoides* is sensitive to climate change, with more suitable temperatures available at higher latitudes. This study provides theoretical guidance for the protection, introduction, and cultivation of *L. macranthoides* under future climate change.

### 3.3. Resource Development and Protection Strategy

*L. macranthoides* is the primary source plant of *Lonicerae Flos*, a precious traditional Chinese medicine that holds economic and scientific research value [6]. The quality of Chinese herbs is intimately linked to their habitat. By analyzing the effect of key environmental variables on the distribution of *L. macranthoides*, suitable areas can be selected for artificial cultivation. This can ensure the quality and yield of *L. macranthoides*, expand its application market, and contribute to the sustainable development of its resources. Meanwhile, this study analyzed the suitable habitats of *L. macranthoides* under current and future climate scenarios to provide a reference for formulating preservation and sustainable development strategies. This study shows that the suitable habitat of *L. macranthoides* will shrink as greenhouse gas emissions increase. This implies that *L. macranthoides* resources will not remain abundant indefinitely. Changes in temperature and precipitation of the driest month may affect the distribution of *L. macranthoides*, while overharvesting of wild populations may also pose a threat. Our model indicated that *L. macranthoides* was mainly distributed in the mountainous areas of southern China, and its wild germplasm resources could be protected from human activities through in situ conservation.

In China, *L. macranthoides* is widely cultivated by farmers due to its ease of cultivation and high economic value. Studies have shown that *L. macranthoides* prefers to grow in open areas with sandy soil, good drainage, neutral pH soil, full sunlight, and moderate altitude [1,3]. A suitable geographical environment contributes to the synthesis of primary and secondary metabolites in *L. macranthoides*, especially indicator ingredients such as chlorogenic acid. Therefore, we recommend that *L. macranthoides* be planted in the mountainous areas of southern China, as these areas feature suitable altitude, sandy soil, good drainage, and full sunlight. Specifically, the suggestions are as follows: (1) protect the wild resources of *L. macranthoides* and establish a germplasm conservation repository; (2) plant *L. macranthoides* on sunny slopes at an elevation of approximately 500 m; (3) improve soil fertility by amending sandy soil with a small amount of clay; (4) regularly maintain drainage ditches to ensure soil permeability. Moreover, insights into the molecular mechanism of combined stress–induced accumulation of bioactive compounds may guide future breeding programs aimed at developing cultivars with consistent quality under changing environmental conditions.

### 3.4. Study Limitations

Our study had several limitations. First, the quality assessment framework for *L. macranthoides* requires further refinement, as its quality should not be determined solely by the three indicator components specified in the pharmacopoeia [7,30]. Therefore, future research should consider adopting a more comprehensive approach to quality evaluation. In addition, the climate data used were derived from various public databases rather than from on–site environmental measurements at the sample collection locations. Therefore, the model results are intended for reference only, and we aim to supplement them with actual observation data in practice.

## 4. Materials and Methods

### 4.1. Collection of Samples and Occurrence Records

From 2024 to 2025, we collected 34 flower samples of *L. macranthoides* from China’s representative producing areas (Table S1), including 13 samples from high–suitable habitats (S1–S13), 10 samples from medium–suitable habitats (S14–S23), and 11 samples from low–suitable habitats (S24–S34). These habitat classifications were defined a priori using a preliminary MaxEnt model before sampling. All samples were identified as *L. macranthoides* by Professor Guoping Peng of Hunan Agricultural University. This study collected 510 species occurrence records from CVH (http://www.cvh.ac.cn/, accessed on 21 March 2025) and NSII (http://www.nsii.org.cn/, accessed on 21 March 2025). Occurrence records with a linear distance of less than 5 km were removed, and ultimately 329 occurrence records of *L. macranthoides* were retained (Figure S1).

### 4.2. Environmental Variables

In this study, 19 bioclimatic and 3 topographic variables were collected from WorldClim 2.1 with a resolution of 2.5 arc–minutes. Fifteen soil variables were collected from the Harmonized World Soil Database v1.2 with a resolution of 30 arc–seconds. Six ultraviolet radiation variables were collected from the gIUV with a resolution of 15 arc–minutes (https://www.ufz.de/gluv/, accessed on 22 March 2025). Two drought index variables were collected from the ET0 Database with a resolution of 30 arc–seconds (https://figshare.com/articles/dataset/Global_Aridity_Index_and_Potential_Evapotranspiration_ET0_Climate_Database_v2/7504448/5, accessed on 22 March 2025). One vegetated surface variable was collected from the GLCNMO with a resolution of 30 arc–seconds (https://globalmaps.github.io/el.html, accessed on 22 March 2025) (Table S6). The “Extract by Mask (Folder)” tool in ArcGIS 10.8 was used to clip the environmental factors. The number of background points in the Maxent model was set to the default value of 10,000. The downloaded environment variable data were unified to 2.5 arc–minutes using ArcMap 10.8 and converted to ASCII format. Paleoclimate data were selected for approximately 22,000 years ago (the Last Glacial Maximum, LGM) and approximately 6000 years ago (the Mid−Holocene, MH). Future climate data were selected from the BCC−CSM model under CMIP6, employing SSP126 (the most optimistic greenhouse gas scenario with radiative forcing stabilizing at 2.6 W·m^−2^ by 2100) and SSP585 (the most pessimistic greenhouse gas scenario with radiative forcing reaching 8.5 W·m^−2^ by 2100) scenarios [15].

To avoid overfitting due to strong correlation and multicollinearity between environmental variables, we performed Pearson correlation analysis using the ‘correlation’ tool in ENMTools v1.4.4. Variables with Pearson’s |r| > 0.8 were excluded [78], and the remaining variables were retained to participate in the predictive distribution (Figure S7). Finally, six climatic variables (Bio_14, Bio_4, Bio_6, Bio_3, Bio_15, and Bio_8), seven soil variables (T_caco3, Awc_−class, S_ph_h2o, T_clay, T_silt, T_oc, and T_ece), three topographic variables (Slope, Elev, and Aspect), two ultraviolet radiation variables (UVB3 and UVB3), two drought index variables (Ai_v3_yr and Et0_v3_yr), and one vegetated surface variable (Gm_lc_v3) were selected (Table S3).

### 4.3. Construction of the Distribution Model

The MaxEnt model was employed to assess the suitable distribution of *L. macranthoides* under various climatic scenarios. 25% of the data were randomly selected for MaxEnt model testing, 75% of the data were utilized for training, and 10 replicate runs were performed with a maximum of 10^6^ iterations. The convergence threshold parameter of the MaxEnt model was set to the default value. The MaxEnt model performance was evaluated using AUC and TSS values [11,15]. The evaluation indices of environmental variables, namely permutation importance and percent contribution, were computed using the jackknife method. The output format of the response curve analysis data was set to Logistic, which was used to determine the optimal ranges of the environmental variables. The maximum test sensitivity plus specificity (MTSPS) was utilized to classify the suitable areas of *L. macranthoides* (Table S7). Meanwhile, the suitable habitat of *L. macranthoides* was classified into four categories, including unsuitable (0−MTSPS), low—(MTSPS−0.3), medium—(0.3–0.5), and high–suitable (0.5–1) habitats [15].

### 4.4. HPLC Analysis

The samples of *L. macranthoides* were dried, pulverized (60 mesh), and stored in a desiccator. A powdered sample of 0.5 g was weighed, and 50 mL of 50% methanol was added for ultrasonic extraction (300 W, 40 kHz). Each group of samples was repeated three times. Chlorogenic acid, macranthoidin B, and dipsacoside B (≥98%, HPLC purity; Solarbio, Beijing, China) were used to establish the standard curve. The standard compounds were dissolved in 50% methanol. Shimazu LC−2030PLUS (Shimadzu, Tokyo, Japan) (chlorogenic acid) and Shimazu LC−16 ELSD (Shimadzu, Tokyo, Japan) (macranthoidin B and dipsacoside B) were utilized to determine the contents of indicator compounds in the samples. A Diamonsil Plus C18 chromatographic column (DIKEMA, Beijing, China) (4.6 × 250 mm, 5 μm) was used. It was 35 °C in the column. Eluent A was acetonitrile, and eluent B was 0.4% acetic acid. The gradient elution program was as follows: 0–10 min, 11.5–15% B; 10–12 min, 15–29% B; 12–18 min, 29–33% B; 18–30 min, 33–45% B; 30–30.1 min, 45–11.5% B. The injection volumes of the Shimazu LC−2030 PLUS and Shimazu LC−16 ELSD were 7 μL and 10 μL, respectively. The flow rate of the machine was set at 1 mL/min, and the detection wavelength was set at 330 nm. In addition, the drift tube temperature was set at 100 °C during the ELSD assay. All standard curves conformed to R^2^ > 0.99 (Figure S8).

### 4.5. HCA and PCA Analysis

In this study, the “systematic clustering” module in SPSS 25 was used for HCA analysis. The PCA analysis was performed utilizing the online platform. The contents of the indicator ingredients were used as variables for HCA and PCA analysis, and the distance matrix among samples was computed. In addition, Pearson’s correlation analysis was resorted to evaluate linear relationships between environmental variables and the content of indicator ingredients.

### 4.6. RNA Extraction and qRT−PCR Validation

Total RNA was extracted from *L. macranthoides* leaves from two treatment groups according to the kit instructions using the StarSpin Plant RNA Kit (GenStar, Beijing, China). One group consisted of plants that grew under a normal outdoor winter environment for one month (113.67° E, 27.98° N, mean temperature 8 °C, low sunshine). The other group consisted of plants grown indoors (room temperature 25 °C, 20,000 lux) for one month. Each group of samples consisted of mixed leaves of four plants with the same maturity stage.

The CDS sequence of phenylpropanoid biosynthesis pathway–related genes was extracted from the genome of *L. macranthoides* via LoniComp (https://www.gzybioinformatics.cn/LoniComp/index.php, accessed on 24 December 2026). The qRT−PCR primers of the above sequences were designed by Primer Premier 6.0, and these genes were amplified (Table S8). cDNA was synthesized using FastKing gDNA RT SuperMix (Tiangen, Beijing, China), and qRT−PCR was performed using Talent qRT−PCR PreMix (Tiangen, Beijing, China) on a ROCGENE Archimed X. *Lm18S* was used as an internal reference gene. Relative gene expression levels were calculated using the 2^−ΔΔCt^ method.

### 4.7. Statistical Analysis

The content of each indicator ingredient was analyzed to determine the mean and standard deviation using Excel 2016. SPSS 25 was used for ANOVA analysis. GraphPad Prism 10 was used for graphing. The statistical analysis was defined as * *p* < 0.05, ** *p* < 0.01, and *** *p* < 0.001.

## 5. Conclusions

This study successfully integrated the MaxEnt model, HPLC, chemometrics, and qRT−PCR to comprehensively assess habitats suitable for *L. macranthoides* in China and to preliminarily reveal the environmental and molecular factors influencing its quality. Key environmental variables influencing distribution included precipitation of driest month, the global aridity index, elevation, temperature seasonality, topsoil carbonate or lime content, slope, mean UV−B of lowest month, and land cover. Currently, suitable habitats are primarily located in southern China between 21° N and 33° N. Under past climate scenarios, the LGM provided more suitable habitats for the distribution of *L. macranthoides*, whereas the MH period witnessed a contraction. Under future projections, increased greenhouse gas emissions are projected to reduce its distribution, but this trend would be mitigated under the SSP126 scenario by 2090 S. Notably, higher contents of chlorogenic acid, macranthoidin B, and dipsacoside B were found in suitable habitats, and these accumulations were associated with soil, altitude, and precipitation. The qRT−PCR results revealed that combined stress (low–temperature and low sunshine) significantly affected the expression of key biosynthetic genes (*LmPAL*, *LmCHS*, *LmCHI*, *LmC4H*, *LmCCoAOMT*, and *LmANS*), providing preliminary molecular evidence of phenylpropanoid pathway regulation by combined stress in *L. macranthoides*. This finding bridges the gap between habitat suitability and phytochemical quality, highlighting the role of environmental stress in shaping secondary metabolism at the transcriptional level. By combining ecological niche modeling, phytochemical profiling, and gene expression analysis, this study establishes a multidisciplinary framework for predicting species distribution and understanding the molecular basis of quality formation under climate change, thereby providing a scientific foundation for the conservation, sustainable cultivation, and stress–oriented breeding of *L. macranthoides*.

## Figures and Tables

**Table 1:** The suitable habitats range of key environmental variables affecting the potential distribution of *L. macranthoides*.

Variable	Total Suitable Habitats Range	Optimum Value	Units
Bio_14	0–190	50.31	mm
Ai_v3_yr	54–54,506	17,740.01	−
Elev	−79–6520	512.27	m
Bio_4	307.041–1735.564	740.17	−
T_caco3	0–15	7.18	% weight
Slope	0–11.853	2.17	°
UVB4	129.596–3000.134	1337.52	J·m^2^·day^−1^
Gm_lc_v3	1–20	12.51	−

## Data Availability

The data generated during this study are available from the first author upon reasonable request.

## Supplementary Materials

The following supporting information can be downloaded at https://www.mdpi.com/article/10.3390/plants15101425/s1. Figure S1. (A) Occurrence records of *L. macranthoides*. (B) Flower of *L. macranthoides* in the field. (C) Dried flower of *L. macranthoides*; Figure S2: ROC curves of the training set and test sets of the model under different climate scenarios; Figure S3: Results of the jackknife test of variables’ contribution to modeling potential distribution of *L. macranthoides*; Figure S4: Response curves of 21 environment variables; Figure S5: The suitable habitat areas of *L. macranthoides* under different climate scenarios; Figure S6: Migration route of *L. macranthoides* in suitable habitat areas in China under climate change in different climate scenarios; Figure S7: (A) Correlation analysis of 19 bioclimatic variables, (B) 15 soil variables, (C) 3 topographic variables, and (D) 6 ultraviolet radiation variables; Figure S8: The standard curve and retention time of chlorogenic acid (A, D), macranthoidin B (B, E), and dipsacoside B (C, E); Table S1: Samples of *L. macranthoides* from different producing areas; Table S2: The AUC and TSS value in various periods of *L. macranthoides* calculated by MaxEnt model; Table S3: Percentage percent contribution and permutation importance of environment variables; Table S4: The suitable habitat areas of *L. macranthoides* under different climate scenarios; Table S5: Centroid coordinates and distances (km) across different time periods (relative to the current period); Table S6: Description of 37 environment variables; Table S7: MTSPS values for different climate scenarios; Table S8: Primers used in qRT−PCR validation.

## Abbreviations

The following abbreviations are used in this manuscript:

LGM	During the Last Glacial Maximum
MH	Mid−Holocene
SSP126	The most optimistic greenhouse gas scenario
SSP585	The most pessimistic
CVH	Chinese Virtual Herbarium
NSII	National Specimen Information Infrastructure
AUC	Area under the subject operating characteristic curve
TSS	True skill statistic
MTSPS	The maximum test sensitivity plus specificity
