# Supplementary material for: Environmental Shaping Suitable Habitats and Quality of Lonicera macranthoides Hand.−Mazz.: Insights from MaxEnt, HPLC, Chemometrics, and Gene Expression Analysis
Source: Plants (Basel). 2026 May 7;15(10):1425. doi: 10.3390/plants15101425 (PMC13210974; doi:10.3390/plants15101425)
Supplement: Supplementary file 1 [file plants-15-01425-s001.zip › Fig S-manuscript.pdf]

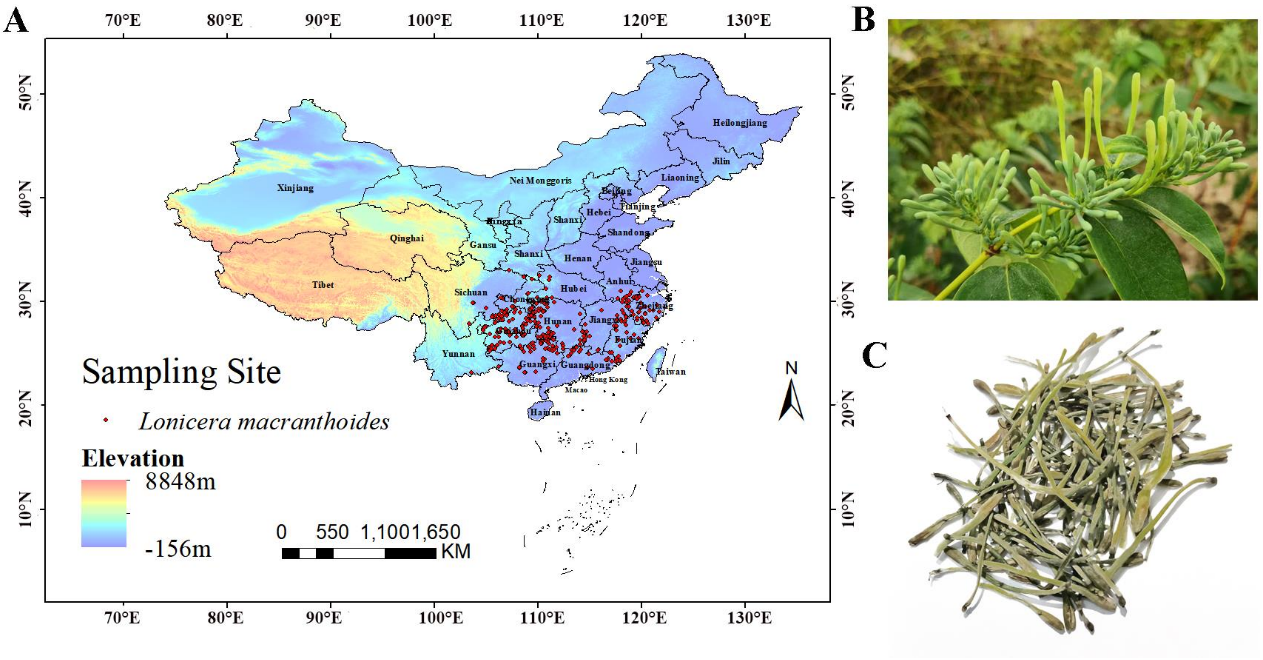

Figure S1. (A) Occurrence records of *L. macranthoides*. (B) Flower of *L. macranthoides* in the field. (C) Dried flower of *L. macranthoides*

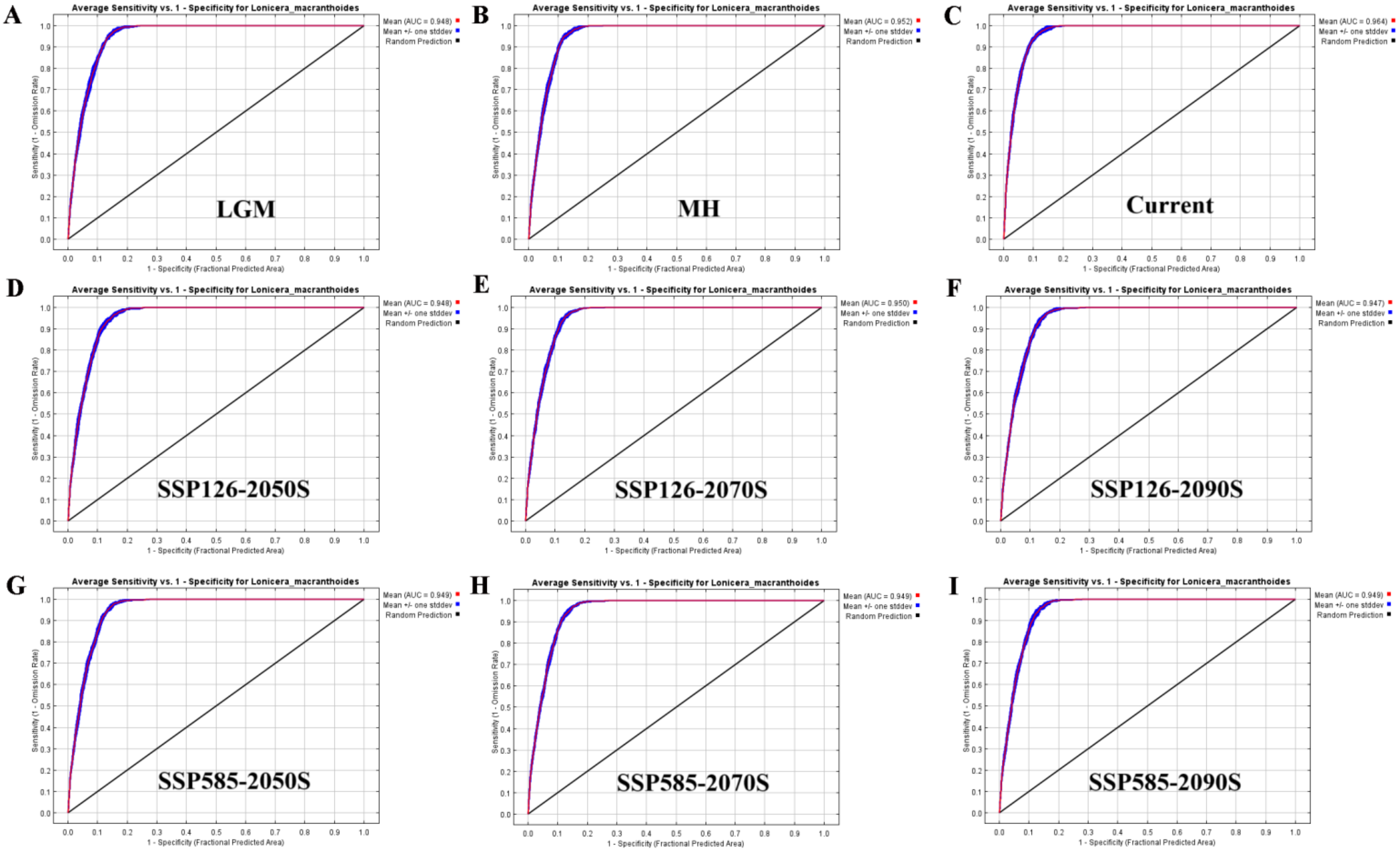

Figure S2. ROC curves of the training set and test sets of the model under different climate scenarios.

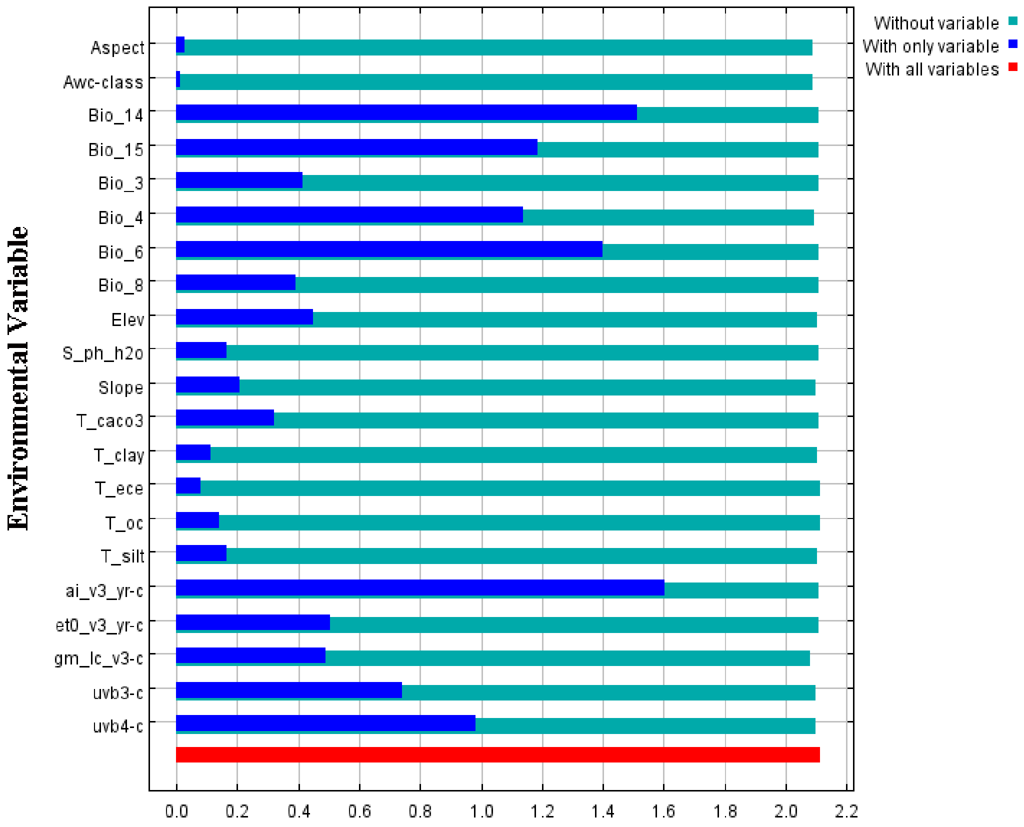

Figure S3. Results of the jackknife test of variables' contribution to modelling potential distribution of *L. macranthoides*.

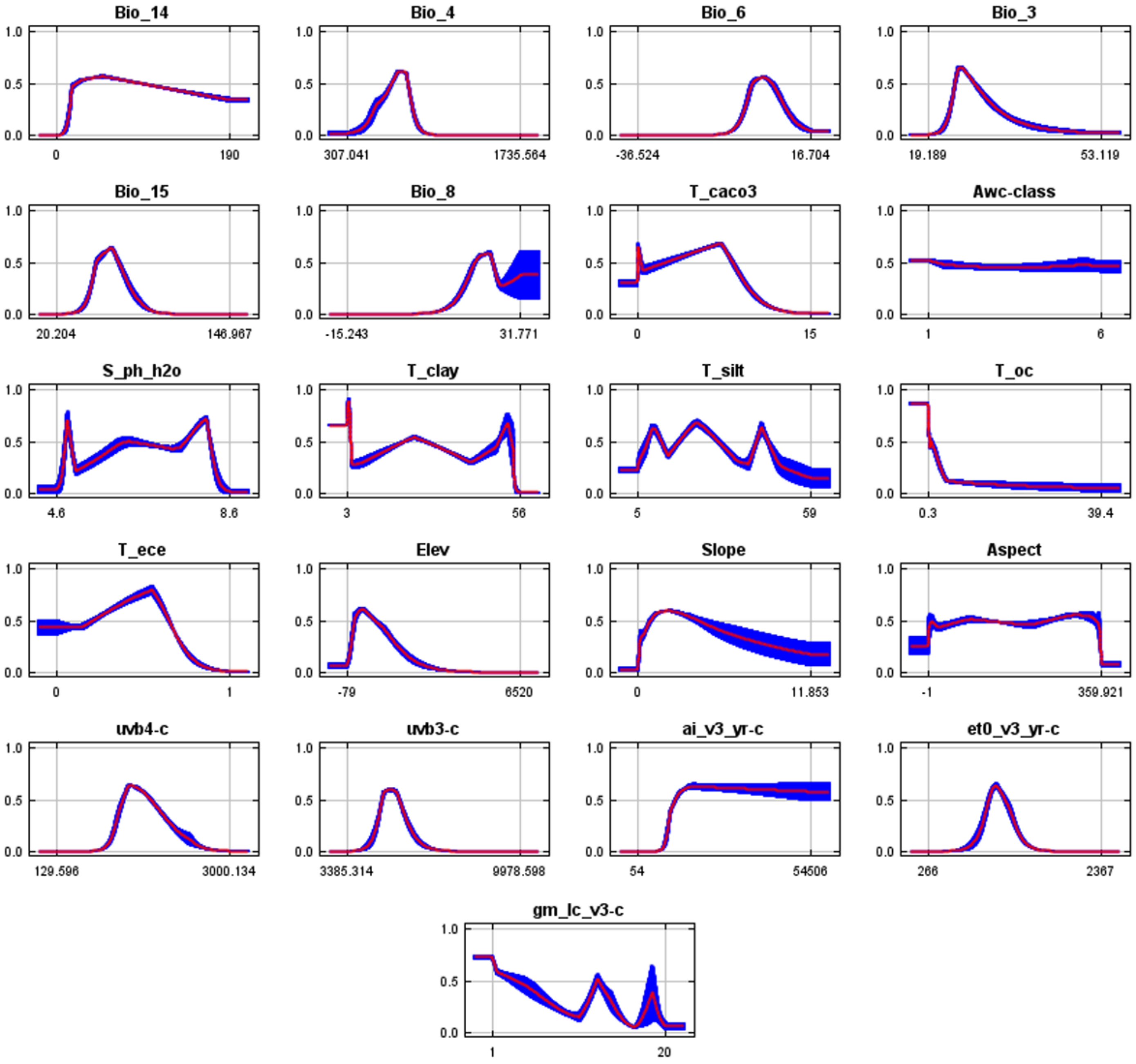

Figure S4. Response curves of 21 environment variables.

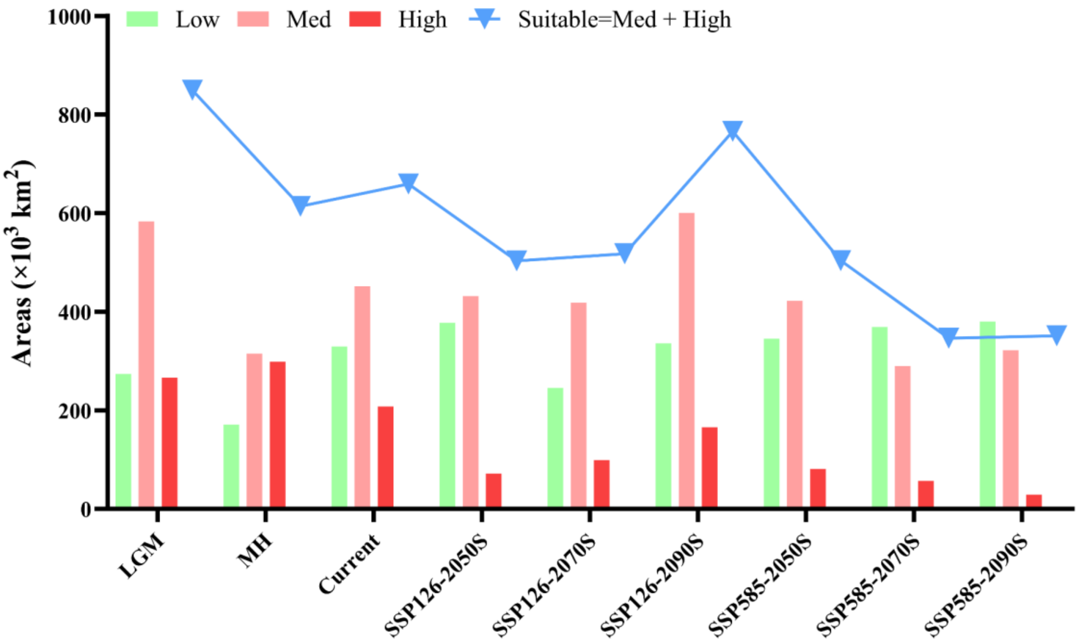

Figure S5. The suitable habitat areas of *L. macranthoides* under different climate scenarios.

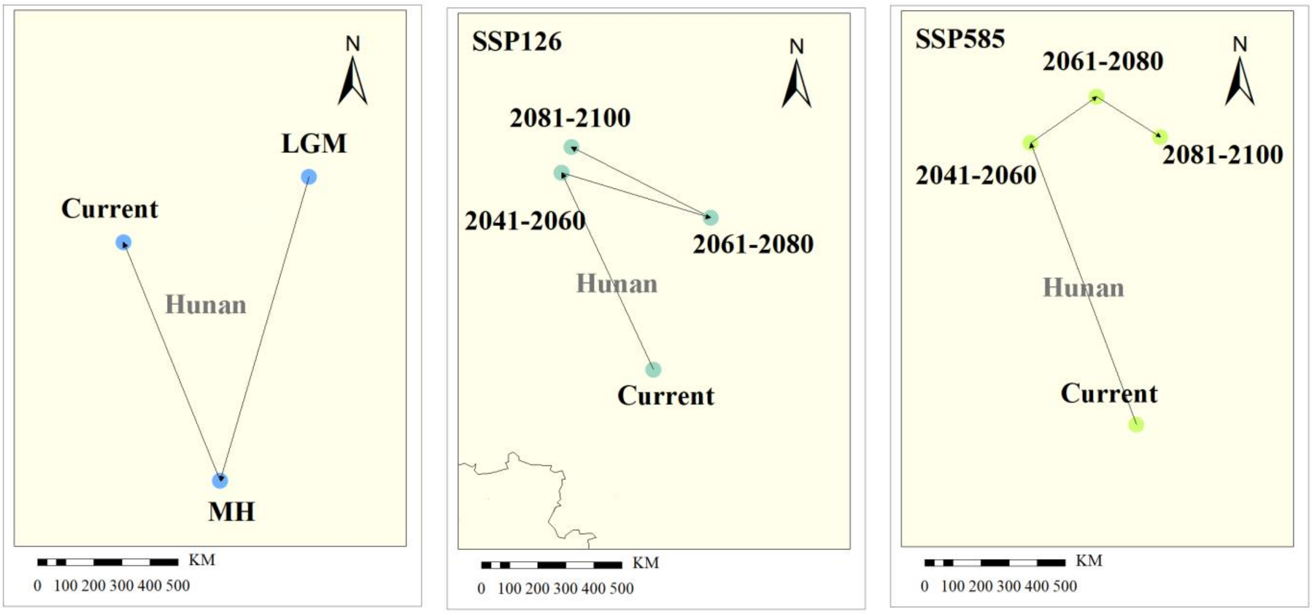

Figure S6. Migration route of *L. macranthoides* in suitable habitat areas in China under climate change in different climate scenarios.

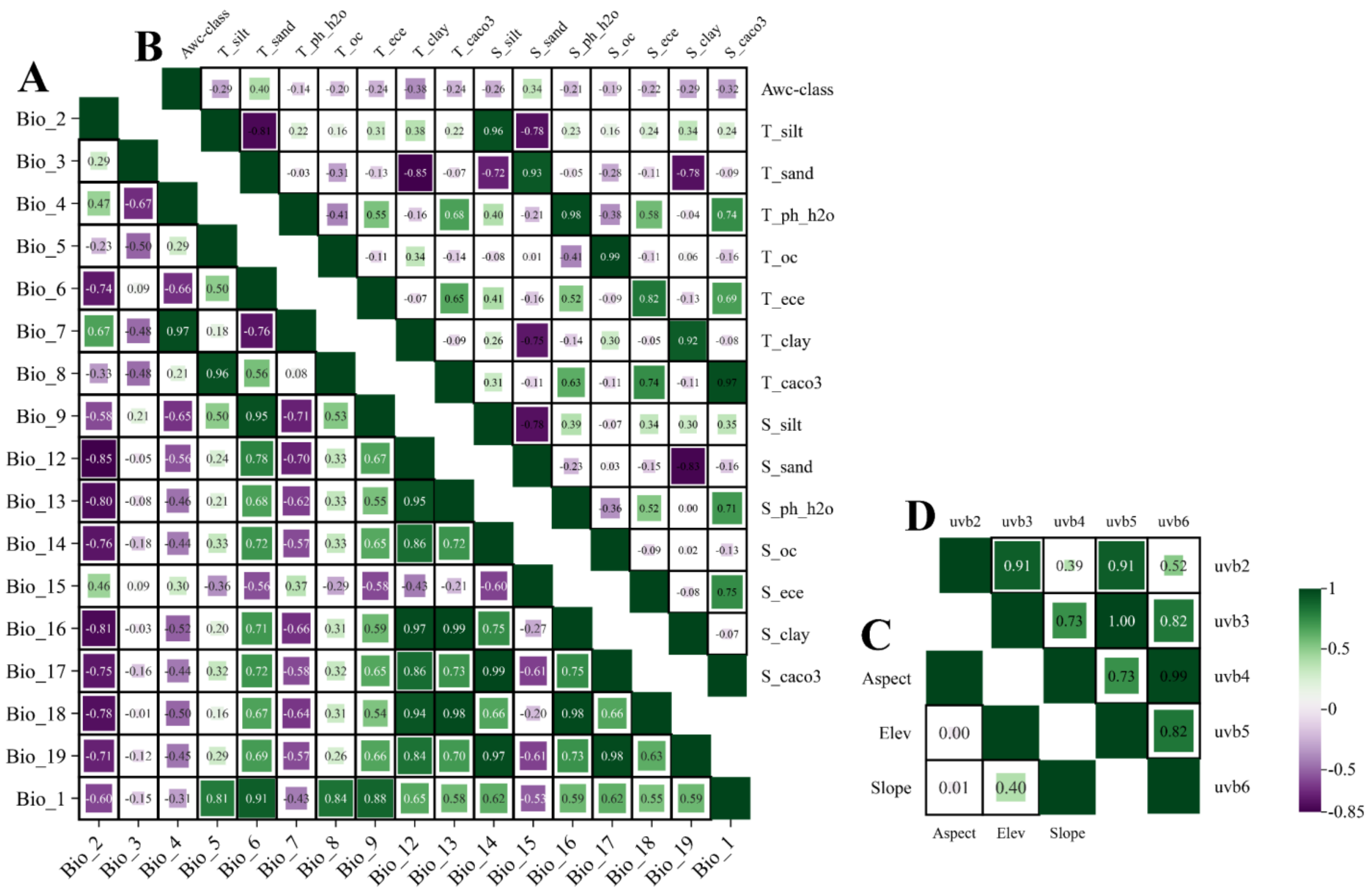

Figure S7. (A) Correlation analysis of 19 bioclimatic variables, (B) 15 soil variables, (C) 3 topographic variables, and (D) 6 ultraviolet radiation variables.

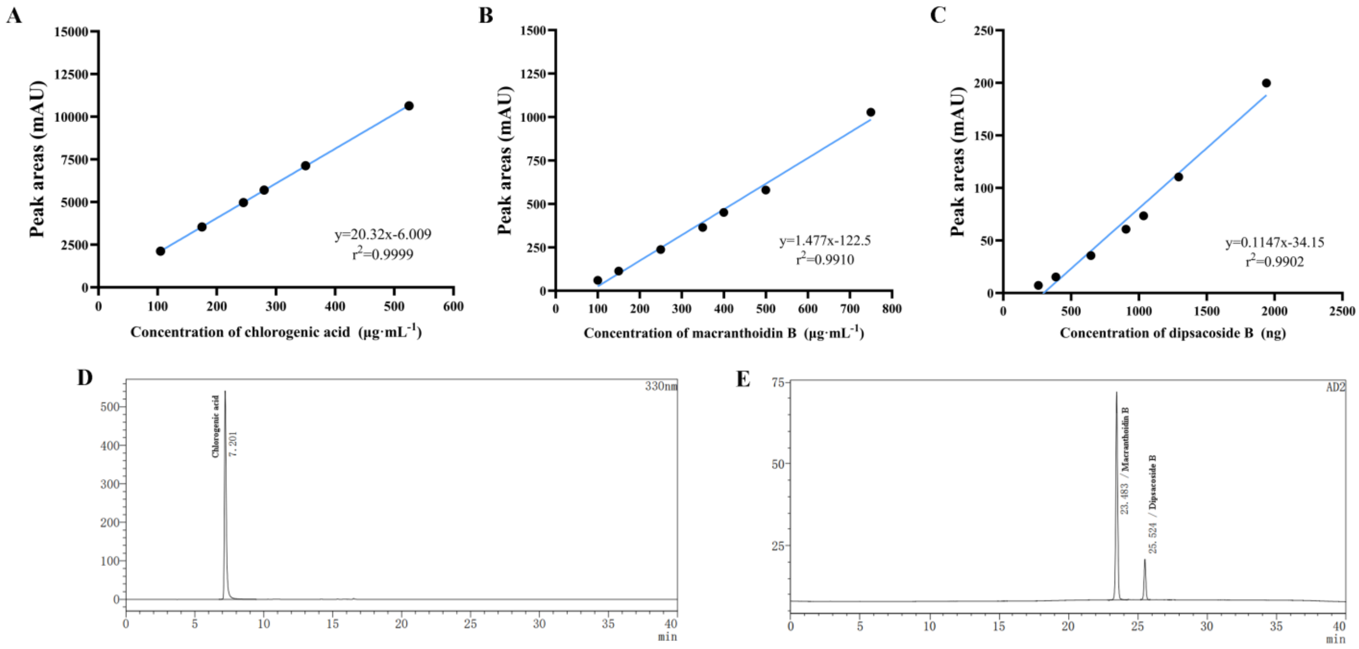

Figure S8. The standard curve and retention time of chlorogenic acid (A, D), macranthoidin B (B, E), and dipsacoside B (C, E).
