# Supplementary material for: Environmental Shaping Suitable Habitats and Quality of Lonicera macranthoides Hand.−Mazz.: Insights from MaxEnt, HPLC, Chemometrics, and Gene Expression Analysis
Source: Plants (Basel). 2026 May 7;15(10):1425. doi: 10.3390/plants15101425 (PMC13210974; doi:10.3390/plants15101425)
Supplement: Supplementary file 1 [file plants-15-01425-s001.zip › Table S-manuscipt.pdf]

Table S1. Samples of *L. macranthoides* from different producing areas.

**Sample No.**	**Location**	Longitude	Latitude	Years
S1	Shaoyang, Hunan, China	110.75° E	27.25° N	4
S2	Shaoyang, Hunan, China	110.75° E	27.55° N	5
S3	Shaoyang, Hunan, China	110.75° E	27.34° N	4
S4	Shaoyang, Hunan, China	110.72° E	27.53° N	5
S5	Shaoyang, Hunan, China	110.73° E	27.51° N	4
S6	Jishou, Hunan, China	109.61° E	28.24° N	3
S7	Xiushan, Chongqing, China	109.17° E	28.48° N	4
S8	Xiushan, Chongqing, China	110.74° E	27.48° N	4
S9	Huaihua, Hunan, China	110.55° E	27.41° N	5
S10	Huaihua, Hunan, China	110.59° E	27.55° N	5
S11	Huaihua, Hunan, China	110.51° E	27.52° N	4
S12	Qianxinan, Guizhou, Chian	105.33° E	25.10° N	5
S13	Qianxinan, Guizhou, Chian	105.37° E	25.09° N	5
S14	Chenzhou, Hunan, China	113.14° E	26.11° N	3
S15	Zunyi, Guizhou, China	107.11° E	27.93° N	4
S16	Zunyi, Guizhou, China	107.29° E	28.23° N	4
S17	Zunyi, Guizhou, China	107.68° E	27.96° N	3
S18	Qiandongnan, Guizhou, China	108.08° E	26.94° N	4
S19	Qiandongnan, Guizhou, China	108.37° E	26.75° N	5
S20	Wuzhou, Guangxi, China	110.98° E	23.11° N	3
S21	Nanning, Guangxi, Chian	108.3° E	23.69° N	5
S22	Wenzhou, Zhejiang, China	120.88° E	28.22° N	4
S23	Shaoguang, Guangdong, China	114.45° E	25.01° N	5
S24	Zhaotong, Yunnan, China	103.34° E	27.38° N	3
S25	Zhaotong, Yunnan, China	103.72° E	27.58° N	4
S26	Bazhong, Sichuan China	107.26° E	31.96° N	4
S27	Bazhong, Sichuan, China	106.97° E	32.26° N	4
S28	Changsha, Hunan, China	113.51° E	28.21° N	3
S29	Changsha, Hunan, China	113.68° E	27.97° N	3
S30	Changsha, Hunan, China	113.68° E	27.96° N	3
S31	Changsha, Hunan, China	113.68° E	27.95° N	3
S32	Jieyang, Guangdong, China	116.11° E	23.32° N	5
S33	Jieyang, Guangdong, China	116.24° E	23.36° N	4
S34	Jian, Jiangxi, China	114.41° E	26.35° N	3

Table S2. The AUC and TSS value in various periods of *L. macranthoides* calculated by MaxEnt model.

Periods	AUC	TSS
Current	0.964	0.842
LGM	0.948	0.851
MH	0.952	0.846
SSP126-2050S	0.948	0.846
SSP126-2070S	0.950	0.849
SSP126-2090S	0.947	0.846
SSP585-2050S	0.949	0.856
SSP585-2070S	0.949	0.851
SSP585-2090S	0.949	0.851

Table S3. Percentage percent contribution and permutation importance of environment variables.

Variable	Percent contribution (%)	Permutation importance (%)
Bio_14	64.2	9
Ai_v3_yr	13.6	6.4
Elev	5.3	3.7
Bio_4	2.9	15.2
T_caco3	2.3	1.4
Slope	2.2	4.1
UVB4	1.8	15.1
Gm_lc_v3	1	3
Aspect	0.9	1.3
Bio_6	0.8	6.6
Awc-class	0.8	2.2
Et0_v3_yr	0.7	5.1
S_ph_h2o	0.7	2.2
UVB3	0.7	10.2
Bio_3	0.5	1.9
Bio_15	0.4	5.6
Bio_8	0.4	2.5
T_clay	0.3	1.5
T_silt	0.3	1.3
T_oc	0.2	0.7
T_ece	0.1	0.9

Table S4. The suitable habitat areas of *L. macranthoides* under different climate scenarios.

Periods		LGM	MH	Current	SSP126-2050S	SSP126-2070S	SSP126-2090S	SSP585-2050S	SSP585-2070S	SSP585-2090S
Areas ($\times 10^3$ km^2)	Low	273.66	171.07	329.44	377.62	245.75	336.04	345.26	369.25	380.12
	Med	582.99	315.13	451.52	431.58	418.36	600.37	422.31	289.79	321.92
	High	266.24	298.68	207.78	71.52	99.21	165.77	81.28	56.76	29.14
	Suitable	849.23	613.81	659.30	503.10	517.57	766.14	503.59	346.55	351.06

Table S5. Centroid coordinates and distances (km) across different time periods (relative to the current period).

Periods	Longitude	Latitude	Distance (km)
Current	111.84°E	26.89°N	-
LGM	112.31°E	27.06°N	50.25
MH	112.08°E	26.29°N	70.84
SSP126-2050S	111.27°E	28.11°N	146.96
SSP126-2070S	112.19°E	27.83°N	110.08
SSP126-2090S	111.33°E	28.27°N	161.46
SSP585-2050S	111.42°E	28.01°N	131.22
SSP585-2070S	111.68°E	28.19°N	145.49
SSP585-2090S	111.93°E	28.03°N	127.07

Table S6. Description of 37 environment variables.

Variable		Description	Units
Bioclimatic variables	Bio_14	Precipitation of Driest Month	mm
	Bio_4	Temperature Seasonality	-
	Bio_12	Annual Precipitation	mm
	Bio_2	Mean Diurnal Range	℃
	Bio_17	Precipitation of Driest Quarter	mm
	Bio_3	Isothermality	1
	Bio_7	Temperature Annual Range	℃
	Bio_18	Precipitation of Warmest Quarter	mm
	Bio_19	Precipitation of Coldest Quarter	mm
	Bio_6	Min Temperature of Coldest Month	℃
	Bio_16	Precipitation of Wettest Quarter	mm
	Bio_15	Precipitation Seasonality	-
	Bio_11	Mean Temperature of Coldest Quarter	℃
	Bio_8	Mean Temperature of Wettest Quarter	℃
	Bio_13	Precipitation of Wettest Month	mm
	Bio_5	Max Temperature of Warmest Month	℃
	Bio_9	Mean Temperature of Driest Quarter	℃
	Bio_10	Mean Temperature of Warmest Quarter	℃
	Bio_1	Annual Mean Temperature	℃
Soil variables	T_caco3	Topsoil carbonate or lime content	% weight
	S_clay	Substratesoil clay content	% weight
	Awc-class	Soil available water content	-
	S_ph_h2o	Substratesoil pH	-
	S_caco3	Substratesoil carbonate or lime content	% weight
	S_oc	Substratesoil organic carbon	% weight
	T_ph_h2o	Topsoil pH	-
	T_clay	Topsoil clay content	% weight
	T_oc	Topsoil organic carbon	% weight
	S_sand	Substratesoil sand content	% weight
	T_silt	Topsoil silt content	% weight
	S_silt	Substratesoil silt content	% weight
	T_ece	Topsoil electroconductibility	s/m
	T_sand	Topsoil sand content	% weight
	S_ece	Substratesoil electroconductibility	s/m
Topographic variables	Slope	Slope	°
	Elev	Elevation	m
	Aspect	Aspect	°
Ultraviolet radiation	UVB1	Annual mean UV-B	$J·m^{2}·day^{-1}$
	UVB2	UV-B seasonality	% per month
	UVB3	Mean UV-B of highest month	$J·m^{2}·day^{-1}$
	UVB4	Mean UV-B of lowest month	$J·m^{2}·day^{-1}$
	UVB5	Sum of monthly Mean UV-B during highest quarter	$J·m^{2}·day^{-1}$

	UVB6	Sum of monthly mean UV-B during lowest quarter	$J \cdot m^2 \cdot day^{-1}$
Drought index	Et0_v3_yr	Standard deviation of annual potential Evapo-transpiration	$mm \cdot day^{-1}$
	Ai_v3_yr	The global aridity index	-
Vegetated surface	Gm_lc_v3	Land cover	-

Table S7. MTSPS values for different climate scenarios.

Periods	MTSPS
Current	0.1813
LGM	0.2004
MH	0.225
SSP126-2050S	0.1787
SSP126-2070S	0.2223
SSP126-2090S	0.2076
SSP585-2050S	0.1991
SSP585-2070S	0.1893
SSP585-2090S	0.2079

Table S8. Primers used in qRT-PCR validation

Gene ID	Primer Sequence
Lm18S	F: AGGCGCGCAAATTACCCAATCC
	R: GCCCTCCAATTGTTCCTCGTTAAG
LmPAL	F: GTCAGGGCTTGCGTCTATTGT
	R: GGTGCTTCAACTTATGCGTCAA
LmCHS	F: TGACCGTGGAGGAGATTCG
	R: ATAGTGAGTAGGAGTCGCTGTT
LmCHI	F: GGGTGAGAGGTTTGGAAATCC
	R: TTGTCGGCGAGTGAAGCA
LmC4H	F: TCTTCTTCGCCGCCGTTATTGC
	R: TTGAGGTCATCGCCGACTTGGA
LmCCoAOMT	F: TTGGCGGTTTGATCGGCTACG
	R: GCTAATACGGCGGCACAAGGT
LmANS	F: CCGTGGAGGAGAAGGAGAAGTA
	R: CAGGGTAGGCAAGGTGAAAGAA
